# Higher serum level of Cystatin C: An additional risk factor of CAD

**DOI:** 10.1097/MD.0000000000024269

**Published:** 2021-01-15

**Authors:** Zhenfei Chen, Jing Zhang, Jun Feng, Gaoliang Zhou, Xiaoqin Jin, Jianyuan Pan

**Affiliations:** a Department of Cardiology, The Second Hospital of Hefei City, Hefei, Anhui, China; b DZHK (German Center for Cardiovascular Research), partner site Heidelberg/Mannheim, Heidelberg University, Germany.

**Keywords:** coronary artery disease, coronary artery lesions, cystatin C, Gensini score

## Abstract

Cystatin C has been proposed as a useful biomarker of early impaired kidney function and a predictor of mortality risk. The present study is to investigate the association between serum Cystatin C and the severity of coronary artery lesions, Gensini score (GS), and the risk of coronary artery disease (CAD).

A total of 682 CAD patients (230 females, 452 males; mean age 62.6 ± 10.7 years, range from 31 to 86 years) and 135 controls (41 females, 94 males; mean age 58.0 ± 10.3 years, range from 38 to 84 years) were recruited in the present study. Enzyme-linked immunosorbent assay was applied to measure serum cystatin C levels and other serum indexes. The estimated glomerular filtration rate and GS were calculated.

Serum low-density lipoprotein cholesterol (LDL-C), uric acid, Cystatin C, and homocysteine (HCY) were significantly elevated in CAD patients compared to controls. There were significant differences regarding total cholesterol, triglyceride, high-density lipoprotein, low-density lipoprotein, cystatin C, eGFR and GS among stable angina pectoris (SAP), unstable angina group (UAP), and acute myocardial infarction (AMI) patients. AMI group had an elevated serum Cystatin C, LDL-C, HCY, and GS than SAP and UAP patients. When stratified patient groups by the quartiles of Cystatin C, we found age, the proportion of male and patients with diabetes, HCY, and GS were increased in Q4 than in other quartile groups. Spearman correlation test revealed a positive relationship between Cystatin C, HCY, and GS. Multivariate logistic regression analysis revealed that serum Cystatin C level, presence of hypertension and diabetes, HCY, age, and male were the risk factors for coronary artery lesions.

In summary, our results suggested that cystatin C is a promising clinical biomarker that provides complementary information to the established risk determinants. The serum Cystatin C level is strongly associated with GS and could be used to evaluate the severity of coronary artery lesions.

## 1 Introduction

Coronary artery disease (CAD) is a prevalent disease and produces immense health and economic burdens all over the world.^[1]^ Cystatin C, a 13-kDa protein, is a member of a family of competitive inhibitors of lysosomal cysteine protease and an alternative endogenous surrogate parameter for assessment of glomerular filtration rate (GFR).^[2]^ Previous studies have proposed cystatin C as a novel biomarker for the evaluation of early impaired renal dysfunction, predominantly to distinguish small decreases in GFR.^[3]^ Of interest, cystatin C has also been proposed as a useful biomarker for cardiovascular risk and is associated with mortality in diverse clinical scenarios.^[4,5]^

Coronary angiography (CAG) was an important tool for the assessment of coronary artery lesions in both clinical practice and scientific investigation in the past.^[6]^ With the development of technology, intravascular ultrasound (IVUS) is another powerful and accurate tool for the evaluation of atherosclerotic burden.^[7]^ The limitation of coronary IVUS and angiography was cost and available; therefore, most CAD registries continue to use angiography to measure atherosclerosis severity.^[8]^ Gensini score (GS), a widely used angiographic scoring system, has been defined and used for evaluation of atherosclerotic burden and prognosis.^[9,10]^ Neeland et al^[11]^ found that the GS system was a valid tool for estimating CAD plaque burden after he compared GS to the IVUS results in 3600 CAD patients.

Multiple clinical and biochemical factors and inflammatory cytokines have been proved to associate with the severity of CAD, such as C1q/TNF-related protein-1,^[12]^ neutrophil-to-lymphocyte ratio and platelets,^[13]^ serum glycated albumin levels,^[14]^ and plasma chemokine.^[15]^ Cystatin C also has been considered an emerging biomarker in cardiovascular disease (CVD) and proved to be an important predictor for adverse outcomes among patients with CAD.^[16,17]^ However, to date, limited research shows the correlation between cystatin C, GS, and coronary artery lesions. Therefore, we conducted the present study to investigate the correlation of serum cystatin C with the severity of coronary artery lesions, GS, and the risk of CAD.

## 2 Method and materials

### 2.1 Study subjects

We performed a prospective cohort study in the Second Hospital of Hefei City from January 2014 to December 2019. This study received ethical approval from the Ethical Committee of the Second Hospital of Hefei City (Hefei, China). Using a convenient sampling method, we selected 682 hospitalized patients diagnosed with CAD (230 females, 452 males; mean age 62.6 ± 10.7 years, range from 31 to 86 years) in the Department of Cardiology at the Second Hospital of Hefei City. 135 control volunteers (41 females, 94 males; mean age 58.0 ± 10.3 years, range from 38 to 84 years) without any symptoms of CAD were included as the control group. Participants with any history of infections, autoimmune diseases, metabolic disease (not including diabetes), and severe chronic diseases (such as cirrhosis, thyroid disease) were excluded. In addition, those with eGFR <60 mL/min or serum creatinine >1.5 mg/dL were excluded from study.

CAG was assessed by 2 independent clinical observers with lesions recorded using a 17-segment modified AHA model.^[18]^ All the patients should fulfill the diagnostic criteria of CAD defined by the American College of Cardiology/American Heart Association in 2014, and were confirmed by CAG to have stenosis ≥50% in at least 1 coronary artery. GS was calculated according to a modified Gensini scoring system. The detailed calculation method was offered by GP Rampidis and showed in his literature.^[19]^

### 2.2 Laboratory investigation

Blood samples were drawn from subjects after an overnight fast before cardiac catheterization. The concentrations of cystatin C were measured by using commercially available Human Elisa Kits (Bio vendor Inc). Blood fasting plasma glucose (FPG), total cholesterol (TC), triglyceride (TG), low-density lipoprotein cholesterol (LDL-C), blood urea nitrogen (BUN), uric acid (UA), homocysteine (HCY), and high-density lipoprotein cholesterol (HDL-C) were determined using an automatic biochemical analyzer (Coulter LX20; Beckman, USA). eGFR was estimated in every patient using the modified MDRD equation (eGFR mL/min/1.73 m^2^ of body surface area186 × [serum creatinine in mg/dL]^−1.154^ × [age in ears]^−0.203^ × [0.742 if female sex]).^[20]^

### 2.3 Statistical analysis

All continuous variables were presented as mean ± SD and frequency for categorical variables. Continuous variables are presented as the means ± standard deviations, the intergroup comparisons were made using the 1-way analysis of variance, and differences between 2 groups were evaluated by Student *t* test. The *χ*^2^ test or Fisher exact test was used to assess differences in categorical data between the 2 groups. We performed multivariate logistic regression analysis to identify the independent risk factors for coronary artery lesions. Spearman rank correlation method was used to determine the correlation between cystatin C levels, HCY, eGFR, and GS.

## 3 Results

### 3.1 Comparison of baseline clinical and laboratory characteristics between healthy control and CAD patients

Baseline demographics, physical examination characteristics, and laboratory characteristics were listed in Table 1. There was significantly higher serum cystatin C, HCY, UA, LDL-C, and FPG in CAD patients than in the control group (all *P* < .05). There was no significant difference in sex and hypertension distribution between CAD patients and controls. CAD patients had a significantly higher age and diabetes proportion and lower serum HDL and eGFR than the control group (all *P* < .05). However, no significant difference was found in BUN, TC, and TG (all *P* > .05).

**Table 1:** Basic clinical characteristic between CAD patients and control groups.

	CAD patients (n = 682)	Control (n = 135)	*P*
Age, y	62.6 ± 10.7	58.0 ± 10.3	**.001**
Males (%)	66.3	69.6	.114
Hypertension (%)	45.7	43.7	.683
Diabetes (%)	20.1	11.1	**.010**
SBP, mmHg	138.4 ± 20.9	120.8 ± 21.8	.341
DBP, mmHg	88.9 ± 62.9	84.4 ± 12.5	.075
FPG, mmol/L	6.4 ± 4.3	5.2 ± 0.2	**.001**
BUN, mmol/L	5.3 ± 1.5	5.2 ± 4.4	.238
UA, μmol/L	379.2 ± 88.6	286.3 ± 78.4	**.001**
HCY, mmol/L	10.7 ± 4.2	9.1 ± 5.1	**.02**
TC, mmol/L	4.2 ± 1.1	4.0 ± 1.1	.053
TG, mmol/L	2.0 ± 1.6	2.0 ± 1.4	.668
HDL-C, mmol/L	1.2 ± 0.6	1.4 ± 0.3	**.003**
LDL-C, mmol/L	2.6 ± 1.1	2.4 ± 0.8	**.010**
eGFR, mL/min/1.73 m^2^	82.6 ± 17.3	92.3 ± 16.7	**.001**
Cys C, mg/L	1.2 ± 0.8	0.8 ± 1.0	**.031**

BUN = blood urea nitrogen, CAD = coronary artery disease, Cys C = cystatin C, DBP = diastolic blood pressure, eGFR = estimating glomerular filtration rate, FPG = fasting plasma glucose, HCY = homocysteine, HDL-C = high-density lipoprotein cholesterol, LDL-C = low-density lipoprotein cholesterol, SBP = systolic blood pressure, TC = total cholesterol, TG = triglyceride, UA = uric acid.

### 3.2 Comparison of baseline clinical and laboratory characteristics among SAP, unstable angina group, and AMI patients

When comparing different variables among different types of CAD patients, the results showed that age, the proportion of male and patients with hypertension or diabetes, TC, LDL-C, HCY, and cystatin C were significantly higher in the acute myocardial infarction (AMI) group than in stable angina pectoris (SAP) group (all *P* < .05). The SAP group had the lowest mean level of cystatin C (1.1 ± 0.9) and AMI group had the highest level (1.6 ± 0.6). HDL-C level and eGFR were significantly higher in the SAP group compared to AMI and unstable angina pectoris (UAP) groups (*P* < .05). However, no statistical differences were found in terms of SBP, DBP, FPG, BUN, UA, and TG (all *P* > .05) (Table 2).

**Table 2:** Comparison of clinical characteristics among different type of CAD patients.

	SAP group (n = 248)	UAP group (n = 237)	AMI group (n = 197)	*P*
Age, y	61.1 ± 10.2	64.6 ± 10.8	65.3 ± 10.0	**.000**
Males (%)	60.5	69.2	75.1	**.002**
Hypertension (%)	56.0	59.9	67.7	**.001**
Diabetes (%)	15.7	23.2	27.8	**.000**
SBP, mmHg	137.6 ± 21.4	139.0 ± 18.8	139.0 ± 22.3	.860
DBP, mmHg	83.2 ± 12.8	93.2 ± 81.7	93.9 ± 89.6	.170
FPG, mmol/L	5.8 ± 2.0	6.6 ± 6.1	6.6 ± 2.8	.320
BUN, mmol/L	5.1 ± 1.5	4.2 ± 1.2	5.4 ± 1.3	.256
UA, μmol/L	289.1 ± 77.1	295.1 ± 82.1	301.2 ± 78.5	.179
HCY, mmol/L	9.1 ± 6.4	10.2 ± 5.5	11.4 ± 4.9	**.03**
TC, mmol/L	3.8 ± 1.1	4.1 ± 1.1	4.4 ± 1.2	**.006**
TG, mmol/L	2.0 ± 1.7	1.8 ± 1.4	2.2 ± 2.0	.113
HDL-C, mmol/L	1.3 ± 0.4	1.2 ± 0.9	1.1 ± 0.3	**.029**
LDL-C, mmol/L	2.1 ± 0.9	2.4 ± 0.8	2.9 ± 1.5	**.000**
eGFR, mL/min/1.73 m^2^	92.2 ± 19.2	84.8 ± 17.5	77.4 ± 20.1	**.032**
Cys C, mg/L	1.1 ± 0.9	1.3 ± 0.7	1.6 ± 0.6	**.030**
Gensini score	17.1 ± 18.9	37.6 ± 31.8	58.3 ± 40.8	**.000**

AMI = acute myocardial infarction group, BUN = blood urea nitrogen, CAD = coronary artery disease, Cys C = cystatin C, DBP = diastolic blood pressure, eGFR = estimating glomerular filtration rate, FPG = fasting plasma glucose, HCY = homocysteine, HDL-C = high-density lipoprotein cholesterol, LDL-C = low-density lipoprotein cholesterol, SAP = stable angina group, SBP = systolic blood pressure, TC = total cholesterol, TG = triglyceride, UA = uric acid, UAP = unstable angina group.

In the UAP and AMI group, the serum cystatin C level and HCY level were decreased with eGFR (*P* < .05). Further analysis showed that serum cystatin C level was positively correlated with HCY level (*r* = 0.65, *P* = .001) and negatively correlated with eGFR (*r* = −0.632, *P* = .031).

### 3.3 Baseline characteristics of CAD patients by cystatin C Quartiles

CAD patients were subdivided in quartiles according to cystatin C plasma concentrations, that is, Q1 < 0.88 mg/L (161 cases); Q2 = 0.88–1.09 mg/L (172 cases); Q3 = 1.09–1.29 mg/L (178 cases); Q4≥1.29 mg/L (171 cases).

When comparing different variables among different groups, the detailed data revealed that the age, proportion of males, and patients with diabetes, HCY, and GS were significantly higher in Q4 than in other groups (all *P* < .05) (Table 3). Of interest, these data showed that a remarkable increase in GS s with an increase of cystatin C. Further analysis showed that serum cystatin C level was positively correlated with the GS (*r* = 0.55, *P* = .021). Neither group showed any significant difference in terms of FBG, TC, TG, HDL, and LDL.

**Table 3:** Baseline characteristics of CAD patients and laboratory variables according to cystatin C concentration quartiles.

	Serum cystatin C levels, mg/L				
Variables	Quartile 1 (<0.88)(n = 161)	Quartile2 (0.88∼1.09)(n = 172)	Quartile 3 (1.09∼1.29)(n = 178)	Quartile 4 (>1.29)(n = 171)	*P*
Age, y	58.0 ± 10.6	61.7 ± 8.5	63.9 ± 10.8	66.6 ± 10.7	**0.000**
Males (%)	56.7	60.6	73.1	74.3	**0.000**
Hypertension (%)	54.7	56.1	63.5	64.3	0.321
Diabetes (%)	13.9	12.6	22.1	31.0	**0.001**
TC, mmol/L	4.0 ± 1.1	4.2 ± 0.97	4.2 ± 1.2	4.2 ± 1.1	0.243
TG, mmol/L	2.0 ± 1.5	1.8 ± 1.3	2.1 ± 1.6	2.1 ± 2.0	0.450
HDL-C, mmol/L	1.2 ± 1.9	1.2 ± 2.4	1.2 ± 1.3	1.2 ± 1.4	0.612
LDL-C, mmol/L	2.5 ± 0.9	2.6 ± 1.2	2.6 ± 1.1	2.7 ± 1.4	0.438
HCY, mmol/L	8.2 ± 5.2	8.7 ± 4.7	9.6 ± 5.1	10.8 ± 4.6	**0.001**
eGFR, mL/min/1.73 m^2^	98.2.2 ± 12.9	91.3 ± 15.7	82.6 ± 13.8	75.8 ± 16.9	**0.000**
Gensini score	23.4 ± 29.3	35.8 ± 30.5	44.8 ± 35.6	59.0 ± 35.9	0.000

CAD = coronary artery disease, DBP = diastolic blood pressure, eGFR = estimating glomerular filtration rate, FPG = fasting plasma glucose, HDL-C = high-density lipoprotein cholesterol, LDL-C = low-density lipoprotein cholesterol, SBP = systolic blood pressure, TC = total cholesterol, TG = triglyceride.

### 3.4 Risk factors for coronary artery lesions

The multivariate logistic regression analysis was used to identify the independent risk factors for coronary artery lesions. The results showed that the risk factors included age (*P* = .042; odds ratio [OR] = 2.33), the proportion of patients with hypertension (*P* = .001; OR = 2.17) and diabetes (*P* = .003; OR = 2.78), and male (*P* = .004; OR = 2.27), as well as HCY (*P* = .03; OR = 1.43) (Table 4).

**Table 4:** Risk factors for coronary artery lesions by multivariate logistic regression analysis.

					95% CI for OR	
	S.E.	Wald	*P*	OR	Lower	Upper
Age	0.21	7.22	.042	2.33	0.40	4.35
Male	0.15	8.51	.004	2.27	1.37	7.14
Hypertension	0.23	11.51	.001	2.17	1.39	3.45
Diabetes	0.27	10.56	.003	2.78	1.61	4.54
HCY	0.131	8.02	.03	1.43	1.12	1.82
Cys C, mg/L						
Q1 (<0.88)				1.00		
Q2 (0.88–1.09)	0.19	1.78	.18	1.28	0.89	1.85
Q3 (1.09–1.29)	0.25	2.91	.09	1.52	0.94	2.49
Q4 (>1.29)	0.21	8.74	.003	2.28	1.24	2.85

CI = confidence interval, Cys C = serum cystatin C, HCY = homocysteine, OR = odds ratio.

For the cystatin C level, the ORs and 95% confidence intervals (CIs) for coronary artery lesions were analyzed by cystatin C quartiles. We adjusted for age, sex, distribution of hypertension and diabetes, and HCY before compared with the first (lowest) quartile. The ORs (95% CI, *P* value) for coronary artery lesions were as follows: second quartile, 1.28 (0.89–1.85, *P* = .18); third quartile, 1.52 (0.94–2.49, *P* = .09); and fourth quartile, 2.28 (1.24–2.85, *P* = .003). These data strongly suggested that increased serum cystatin C levels were related to the severity of coronary artery lesions.

## 4 Discussion

This cross-sectional study of 682 China's patients with CAD or 135 controls revealed the relationship between serum cystatin C level and the risk of CAD, the severity of atherosclerotic burden of the coronary arteries. Other interesting findings of the present study were the fact that cystatin C levels were not only negatively associated with eGFR but also significantly and positively related to the GS. The high serum levels of cystatin C were associated with a high score of GS, which means higher severity of atherosclerotic burden of the coronary arteries.

Since originally discovered, cystatin C was linked through countless studies with renal function. Renal dysfunction was recognized as an independent risk factor for CVD.^[21]^ Researchers have proved that patients with chronic kidney dysfunction are at a higher risk of CVD compared to the general population, and show a higher rate of cardiovascular events/mortality.^[22]^ Past studies have disclosed the close relationship between high serum level of cystatin C and stable CAD,^[5]^ acute coronary syndrome, non-ST-segment elevation acute coronary syndrome,^[23]^ and ST-segment elevated myocardial infarction.^[24]^ Our results are consistent with past studies, here we also proved that the severity of CAD and worse clinical outcome were related to high cystatin C levels.^[25–27]^ Thus, the level of cystatin C could be a promising clinical biomarker for predicting the severity of CAD.

The association between GS and prognosis of CAD such as mortality and disease progression has been demonstrated in previous studies.^[28–30]^ Unfortunately, few researchers put their attention to the role of cystatin C in predicting the severity of atherosclerotic plaque burden of the coronary arteries. Woitas et al^[30]^ analyzed data from 2998 patients of the LURIC (Ludwigshafen Risk and Cardiovascular Health) study with a median follow-up of 9.9 years and a strong relationship was revealed between the concentration of Cystatin C and long-term all-cause and cardiovascular mortality. In another multicenter trial, Keller et al^[27]^ found that the mortality of CAD was increased with elevated cystatin C levels in 2162 patients. The association between a high level of cystatin C and the risk of mortality and morbidity had also been revealed in other different cohorts.^[31–34]^ Our present study results strongly implied that the serum level of cystatin C may be a promising biomarker for the evaluation of atherosclerotic plaque burden.

Till now, the detailed mechanism of cystatin C affects the progression of CAD is still unclear. The following factors could be used to explain the mechanism. First, cystatin C was a cysteine protease inhibitor that was produced at a constant rate by all nucleated cells and freely filtered by glomeruli owing to its low molecular weight. Therefore, cystatin C was not only a useful marker in judging the early renal insufficient but also could be an important tool for predicting the long-term prognosis of renal dysfunction.^[35,36]^ Several studies have revealed that early renal dysfunction was a strong and independent risk of CAD.^[37,38]^ Systemic micro-inflammation response or oxidative stress would accelerate the progression of atherosclerosis in CAD patients with early renal impairment.^[39,40]^ The risk of CAD Patients with a high level of cystatin C was much higher than those with a normal level of Cystatin C, suggesting those patients had preexisting renal insufficiency. Secondly, past studies have proved that smoking, obesity, and chronic inflammation affect the production of cystatin C; all those factors were risk of CAD.^[41–43]^ In our present study, a high serum level of cystatin C was associated with a high GS, suggesting that cystatin C had an active role in the biological process leading to format atherosclerotic plaque. Thirdly, cystatin C was a key endogenous cathepsin inhibitor and its main function was to keep the balance between proteases and their main inhibitor.^[44]^ Increased serum levels of cystatin C can lead to break the balance between cystatin C and cysteine cathepsins. Thus, atherosclerosis formation and development will be further accelerated in CAD patients.^[45,46]^

Several limitations should be acknowledged in the present study. First, the present study was a hospital-based observational study, of which study subjects participated in our study came for medical treatment; these patients paid more attention to their health, and this may lead to a selection bias. Second, only CAD patients were included in the present study, so our results cannot be applied to the other study population. Third, cystatin C levels were measured only once; we could not discriminate whether an elevated cystatin C level was influenced by acute kidney injury or chronic kidney disease. Furthermore, the sample size was relatively small in our study, especially for the control group. The small sample size may reduce the statistical power and cause a contradictory result. Further well-designed and follow-up studies with larger samples are still needed to determine the precise relationships between cystatin C and CAD.

In summary, our results suggested that the serum cystatin C level was strongly associated with the GS and it can be used to evaluate the severity of CAD. An elevated cystatin C concentration could be an independent factor associated with the progression of CAD and be a useful clinical marker that provides complementary information to the established risk determinants.

## Acknowledgments

The authors thank all participants for their technical assistance and advice regarding statistical analysis.

## Author contributions

**Data curation:** jun feng, Gaoliang Zhou, Xiaoqin Jin.

**Formal analysis:** jing zhang.

**Supervision:** Jianyuan Pan.

**Writing – original draft:** zhenfei chen.

**Writing – review & editing:** Jianyuan Pan.

## Abbreviations

Abbreviations: AMI = acute myocardial infarction, CAD = coronary artery disease, Cys C = Cystatin C, eGFR = estimated glomerular filtration rate, GS = Gensini score, HCY = homocysteine, HDL-C = high-density lipoprotein cholesterol, LDL-C = low-density lipoprotein cholesterol, SAP = stable angina pectoris, TC = total cholesterol, UAP = unstable angina pectoris.
